# Predictors of virologic outcome among people living with HIV who continue a protease inhibitor-based antiretroviral regimen following virologic failure with no or limited resistance

**DOI:** 10.1186/s12981-022-00494-9

**Published:** 2023-01-05

**Authors:** Robert A. Salata, Beatriz Grinsztejn, Justin Ritz, Ann C. Collier, Evelyn Hogg, Robert Gross, Catherine Godfrey, Nagalingeswaran Kumarasamy, Cecilia Kanyama, John W. Mellors, Carole L. Wallis, Michael D. Hughes, the ACTG A5288 Study Team

**Affiliations:** 1 Case Western Reserve University, 11100 Euclid Avenue, Cleveland, OH 44122 USA; 2 Instituto Nacional de Infectologia Evandro Chagas, Rio de Janeiro, Brazil; 3 Harvard T.H. Chan School of Public Health, Boston, MA USA; 4 University of Washington, Seattle, WA USA; 5 Social & Scientific Systems, A DLH Company, MD Silver Spring, USA; 6 University of Pennsylvania Perelman School of Medicine, Philadelphia, PA USA; 7 NIAID, Bethesda, MD USA; 8 YR Gaitonde Center for AIDS Research and Education, Chennai, India; 9 University of North Carolina Project–Malawi, Lilongwe, Malawi; 10 University of Pittsburgh, Pittsburgh, PA USA; 11 Lancet Laboratories and BARC SA, Johannesburg, South Africa

## Abstract

**Background:**

Treatment management after repeated failure of antiretroviral therapy (ART) is difficult due to resistance and adherence challenges. For people who have failed non-nucleoside reverse transcriptase inhibitor-(NNRTI-) and protease inhibitor-(PI-) based regimens with no or limited resistance, remaining on PI-based ART is an option. Using data from an ART strategy trial (A5288) in low/middle-income countries which included this option, we explored whether predictors can be identified distinguishing those who experienced further virologic failure from those who achieved and maintained virologic suppression.

**Methods:**

A5288 enrolled people with confirmed HIV-1 RNA ≥ 1000 copies/mL after ≥ 24 weeks of PI-based ART and prior failure on NNRTI-based ART. This analysis focused on the 278 participants with no resistance to the PI being taken and no or limited nucleoside reverse transcriptase inhibitor (NRTI) resistance, who continued their PI with flexibility to change NRTIs. Proportional hazards models were used to evaluate predictors of virologic failure during follow-up (VF: confirmed HIV-1 RNA ≥ 1000 copies/mL at ≥ 24 weeks of follow-up).

**Results:**

56% of participants were female. At study entry, median age was 40 years, time on ART 7.8 years, CD4 count 169 cells/mm^3^, HIV-1 RNA 20,444 copies/mL; and 37% had NRTI resistance. The estimated proportion experiencing VF increased from 39% at week 24 to 60% at week 96. In multivariable analysis, significant predictors at study entry of VF were higher HIV-1 RNA (adjusted hazard ratio: 2.20 for ≥ 10,000 versus < 10,000 copies/mL), lower age (1.96 for < 30 versus ≥ 30 years), NRTI resistance (1.74 for present versus absent), lower CD4 count (1.73 for < 200 versus ≥ 200 cells/mm^3^), and shorter ART duration (1.62 for < 10 versus ≥ 10 years). There was a strong trend in proportion with VF at week 96 with the number of these five risk factors that a participant had, varying from 8% for zero, to 31%, 40%, 73%, and 100% for one, two, three, and four/five. Only 13% of participants developed new NRTI or PI resistance mutations.

**Conclusion:**

A simple count of five predictors might have value for identifying risk of continued VF. Novel antiretroviral and adherence support interventions are needed to improve virologic outcomes for higher risk individuals.

**Supplementary Information:**

The online version contains supplementary material available at 10.1186/s12981-022-00494-9.

## Introduction

Even with the advent of dolutegravir-based antiretroviral therapy (ART), increasing numbers of people living with HIV in low- and middle-income countries (LMICs) will receive ART including a protease inhibitor (PI) following virologic failure on ART which included a non-nucleoside reverse transcriptase inhibitor (NNRTI) [1, 2]. People in LMICs who have experienced treatment failure on both NNRTI- and PI-based ART demonstrate a wide array of genotypic resistance mutations or wild type virus possibly due to adherence challenges [3, 4]. For those people who have experienced failure but who show no or limited resistance, remaining on a PI-based regimen with enhanced adherence support is an option [5, 6]. However, to better inform the decision to pursue this option, there is a need to identify possible predictors of ongoing virologic failure versus potential for virologic suppression if the existing regimen is continued.

The AIDS Clinical Trials Group (ACTG) conducted a clinical trial of a treatment strategy for individuals failing second-line PI-based ART in LMICs (study A5288 [NCT01641367] [7]). The study used genotyping to characterize each individual’s resistance profile including identifying a cohort (“Cohort A”) with no lopinavir/ritonavir (LPV/r) resistance and susceptibility to at least one NRTI who continued on PI-based ART during follow-up, while individuals who had higher levels of resistance were assigned to other cohorts and switched to third-line ART which included newer antiretrovirals (darunavir/ritonavir, etravirine and raltegravir). Cohort A comprised about half of participants in the A5288 study population. In the primary results of the study, with continuation of a PI-based regimen, approximately half of participants in Cohort A achieved and maintained virologic suppression [7]. Nevertheless, this meant that approximately half experienced further virologic failure. Our objective was to identify demographic and clinical predictors of virologic failure among individuals in Cohort A who continued PI-based ART.

## Methods

### Study design and participants

The A5288 study was an open-label phase IV, interventional trial evaluating an ART strategy for individuals experiencing virologic failure on their second-line PI-based regimen at 19 urban clinical sites in 10 countries in Africa (Kenya, Malawi, South Africa, Uganda and Zimbabwe), Latin America (Brazil, Haiti, and Peru) and Asia (India and Thailand). The design has previously been described in detail [7, 8]. Key eligibility criteria included people aged ≥ 18 years living with HIV previously treated with an NNRTI-based regimen replaced by a PI-based regimen, with the change due to toxicity or virologic failure. Participants had to be experiencing virologic failure defined as two consecutive measurements of plasma HIV-1 RNA ≥ 1000 copies/mL obtained at least one day apart after at least 24 weeks on the same PI-based regimen.

Ethical approvals were obtained from the institution review boards at all the participating clinical sites, as follows: Case Western Reserve University, Instituto Nacional de Infectologia Evandro Chagas, Harvard Longwood Campus (HLC) Institutional Review Board, University of Washington, Joint Clinical Research Centre, University of Pennsylvania Perelman School of Medicine, National Institutes of Health, YR Gaitonde Center for AIDS Research and Education, University of North Carolina, National Commission for Science and Technology Malawi, University of Pittsburgh, and the National Health Research Ethics Council (NHREC) (South Africa). Written informed consent was obtained from participants before undertaking study procedures.

### Procedures

Real-time HIV genotype resistance results, treatment history and, if available, any historical resistance results, were used to assign participants to one of four treatment cohorts (A, B, C, or D) [7]. Site investigators made a decision about cohort and regimen recommendations with final adjudication by the A5288 Clinical Management Team. Participants in Cohort A had no lopinavir/ritonavir (LPV/r) resistance and susceptibility to at least one NRTI. They continued on their existing PI during study follow-up but could have their NRTIs modified, including use of study-provided combination emtricitabine/tenofovir disoproxil fumarate (FTC/TDF). Analyses in this report focus on Cohort A but we excluded nine participants who had resistance to the PI being continued or to both NRTIs (because of minor resistance to atazanavir [ATV], the PI they continued; toxicity to the susceptible NRTI; or genotyping sample mix-up) (Additional file 1: Fig. S1). Thus all participants included had no resistance to the PI being continued, and had susceptibility to at least one of the NRTIs taken during follow-up.

All participants were followed until the last enrolled participant reached 48 weeks of follow-up. Study visits occurred at weeks 4, 12, 24 and every 12 weeks thereafter, with HIV-1 RNA measurements at weeks 12, 24 and every 24 weeks thereafter. Participants with HIV-1 RNA ≥ 1000 copies/mL during follow-up had an additional HIV-1 RNA test as soon as possible (ideally within 4 weeks) and collection of a plasma sample for possible genotyping. In the event of confirmed virologic failure (two consecutive HIV-1 RNA ≥ 1000 copies/mL at or after 24 weeks), a genotype resistance test was performed, and participants were assigned to an ART regimen using the same approach as at study entry. Adherence counseling by site staff occurred at study pre-entry and entry visits, and then adherence support followed the site’s standard of care (SOC). Except in Peru, participants at all sites were randomized to receive either a cellphone adherence support intervention (CPI) in addition to SOC adherence support (CPI + SOC) or SOC adherence support. The CPI was used for the first 48 weeks of follow-up. Results comparing virologic and other outcomes for CPI + SOC versus SOC have been previously reported and showed no significant effect of CPI [8].

### Outcomes

This report focuses on the key secondary outcome measure in the A5288 study of time from study entry to confirmed virologic failure, defined as the time to the first of two successive HIV-1 RNA measurements ≥ 1000 copies/mL at or after 24 weeks from study entry. One participant who died following an HIV-1 RNA measurement ≥ 1000 copies/mL at 24 weeks without confirmation was considered as having confirmed virologic failure. Among those experiencing virologic failure during follow-up, we further considered whether or not the resistance genotype obtained at virologic failure showed new resistance-associated mutations according to the Stanford HIV Database algorithm version 6.2 [7, 9] compared with the genotype obtained during screening for the study.

### Statistical analysis

This is an exploratory analysis of predictors of time to confirmed virologic failure. The following variables assessed at study entry were considered as possible predictors: sex, age (initially categorized as < 30, 30–39, 40–49 or ≥ 50 years), HIV-1 RNA at study entry (< 10,000, 10,000 – <100,000, ≥ 100,000 copies/mL), CD4 count (≤ 50, 51–199, ≥  200 cells/mm^3^), duration of time since first starting ART (defined by quartiles: ≤ 5.5, > 5.5 – ≤ 7.8, > 7.8 – ≤ 9.9, > 9.9 years) any level of resistance using the Stanford HIV Database algorithm version 6.2 to any NRTI commonly used at the time in LMICs (yes or no, specifically to lamivudine [3TC], didanosine [DDI], stavudine [D4T], abacavir [ABC], zidovudine [ZDV], FTC and TDF), and the randomized adherence support intervention (CPI + SOC, SOC). Kaplan-Meier estimates and proportional hazards models were used to evaluate associations of these variables with time to confirmed virologic failure, with Gray’s test used to test associations involving the competing risks of virologic failure with, and without, new resistance mutations. Analysis was on an intent to continue treatment basis, i.e. ignoring any changes in antiretroviral treatment. For participants not experiencing virologic failure, follow-up was censored at the last available HIV-1 RNA measurement. Associations were considered significant if p < 0.05.

## Results

A total of 287 participants were enrolled into Cohort A between 2013 and 2015, representing 53% of the overall A5288 study population [5]. As described in the Methods, we excluded nine participants, giving 278 participants in the study population for this report. Table 1 describes the characteristics at study entry of these 278 participants: 56% were female and median age was 40 years. At study entry, median time on ART was 7.8 years, median time on a PI-based regimen was 3.2 years, and median CD4 count was 169 cells/mm^3^. Median HIV-1 RNA was 20,444 copies/mL and 52 (19%) had HIV-1 RNA < 1000 copies/mL at study entry despite having confirmed HIV-1 RNA ≥ 1000 copies/mL during screening.

**Table 1:** Characteristics of participants at study screening and study entry, and antiretroviral regimens received during follow-up

Characteristic		Total (N = 278)
Age at entry (years)	Median (IQR)	40 (34, 46)
	10th, 90th percentiles	22, 55
Sex	Male	123 (44%)
	Female	155 (56%)
Country	Brazil	25 (9%)
	Haiti	33 (12%)
	India	50 (18%)
	Kenya	32 (12%)
	Malawi	17 (6%)
	Peru	18 (6%)
	South Africa	56 (20%)
	Thailand	7 (3%)
	Uganda	34 (12%)
	Zimbabwe	6 (2%)
HIV-1 RNA at entry (copies/mL)	Median (IQR)	20,444 (2,015, 91,371)
	10th, 90th percentiles	272, 307,315
CD4 count at entry (cells/mm³)	Median (IQR)	169 (72, 288)
	10th, 90th percentiles	23, 431
HIV-1 Subtype	A1	49 (18%)
	B	73 (26%)
	C	130 (47%)
	D	12 (4%)
	CRF01_AE	8 (3%)
	Other	6 (2%)
Duration of ART Prior to Entry (years)	Median (IQR)	7.8 (5.5, 9.9)
	10th, 90th percentiles	3.6, 11.8
Duration of PI-based ART from last NNRTI use (years)	Median (IQR)	3.2 (1.8, 5.0)
	10th, 90th percentiles	1.3, 7.9
NRTI resistance at screening	Resistance to any NRTI	104 (37%)
	Susceptible	174 (63%)
NNRTI resistance at screening	Resistance to any NNRTI	146 (53%)
	Susceptible	132 (47%)
PI resistance at screening	Resistance to any PI	20 (7%)
	Susceptible	258 (93%)
Randomized adherence support intervention	CPI + SOC	128 (46%)
	SOC	132 (47%)
	Did not participate	18 (6%)
NRTI components of regimen during follow-up	3TC,ABC	1 (< 1%)
	3TC,TDF	6 (2%)
	3TC,ZDV	20 (7%)
	FTC,TDF	250 (90%)
	FTC,TDF,ZDV	1 (< 1%)
PI component(s) of regimen during follow-up	ATV	1 (< 1%)
	RTV-boosted ATV	103 (37%)
	RTV-boosted LPV	173 (62%)
	SQV	1 (< 1%)

Drugs included in the interpretation of class resistance for NRTIs were: lamivudine (3TC), emtricitabine (FTC), abacavir (ABC), zidovudine (ZDV), stavudine (d4T), didanosine (ddI) and tenofovir (TDF)

Drugs included in the interpretation of class resistance for NNRTIs were: efavirenz (EFV), nevirapine (NVP), etravirine (ETR) and rilpivirine (RPV)

Drugs included in the interpretation of class resistance for PIs were: atazanavir/r (ATV/r), darunavir/r (DRV/r), indinavir/r (IDV/r), lopinavir/r (LPV/r), nelfinavir (NFV), saquinavir/r (SQV/r), tipranavir/r (TPV/r), and fosamprenavir/r (FPV/r). Note though that the cohort studied was selected on the basis that participants had no resistance to LPV/r and, if receiving ATV/r, no resistance to ATV/r

Two sites in Peru did not participate in the randomized adherence support intervention and therefore the participants are indicated as ‘Did not participate’

The PI continued was LPV/r for 173 participants (62%) and ATV/ritonavir (ATV/r) for 103 (37%); for two other participants it was ATV (because of intolerance to ritonavir) or saquinavir (SQV). All participants took either 3TC or FTC. The second NRTI was TDF for 256 participants (92%), ZDV for 20 participants (7%), and ABC for one participant (< 1%); the remaining participant (< 1%) took both TDF and ZDV (plus FTC). Of the 278 participants, 104 (37%) had NRTI resistance based on the genotype obtained at screening including 89 (32%) with resistance to one NRTI in the regimen taken during follow-up (or to two NRTIs for the participant who took both TDF and ZDV plus FTC). Thus 189 participants (68%) had virus susceptible to both the two NRTIs and the PI taken. Among those with resistance to the NRTI component on the regimen taken, 80 participants (29% of the study population of 278 participants) had high-level resistance to 3TC/FTC (due to the M184V mutation), two (< 1%) had intermediate-level resistance to 3TC/FTC (one of the two took TDF and ZDV plus FTC also had intermediate resistance to TDF), and seven (3%) had low-level resistance or potential low-level resistance to the other NRTI (which was to TDF for all seven) (Additional file 1:  Table S1).

Retention was very high: during median follow-up of 72 weeks (quartiles: 60, 96), only 6 participants (2%) were lost to follow-up without first experiencing virologic failure. Only 12 (4%) of participants had a change in their ART regimen prior to virologic failure or prior to their last HIV-1 RNA measurement if they did not experience virologic failure. Six changed due to adverse events, four due to noncompliance or participant decision to stop ART, one due to a drug supply issue, and one due to a potential drug interaction with tuberculosis treatment.

**Fig. 1:**
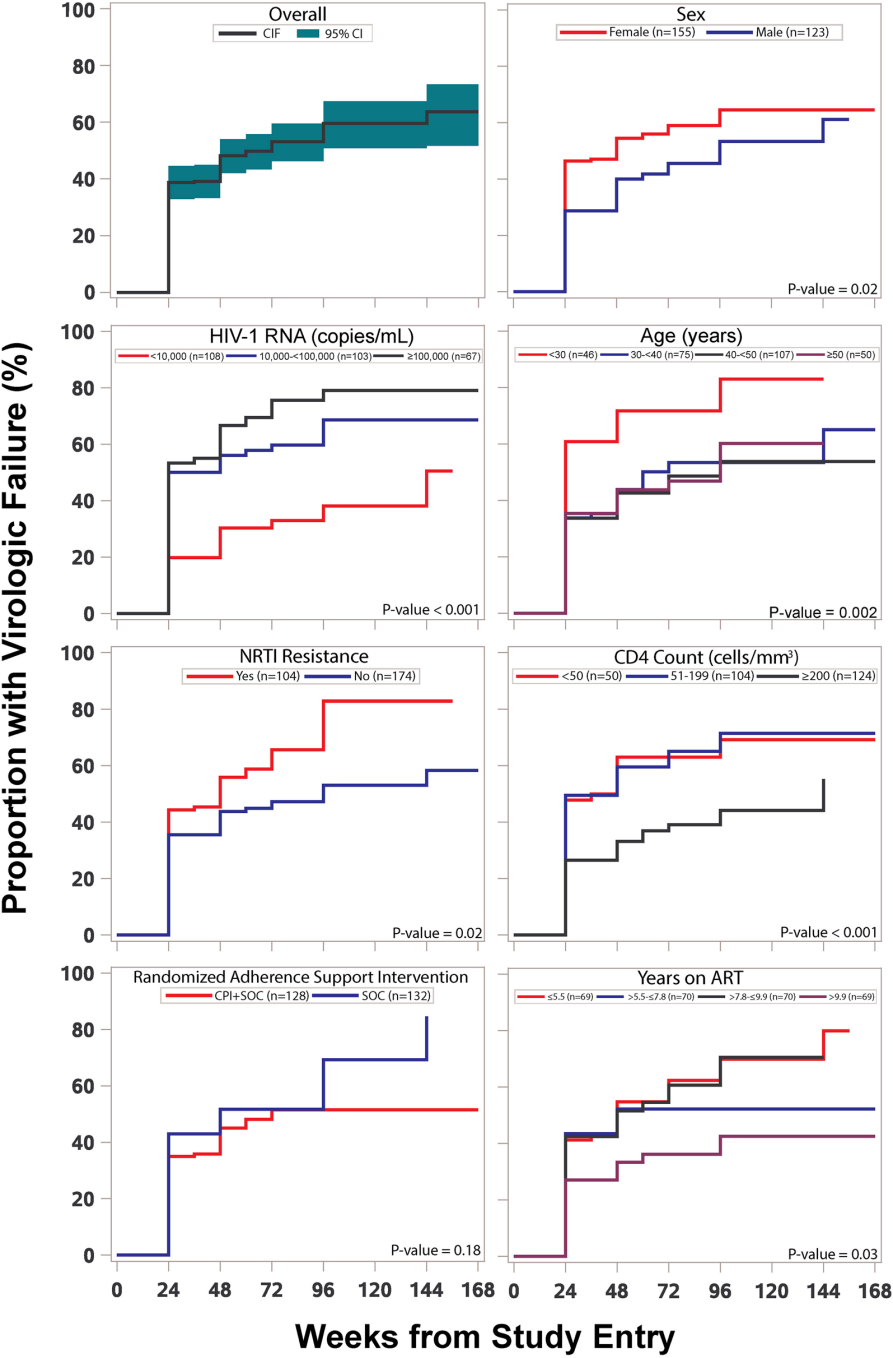
Estimated Cumulative proportion of participants with virologic failure (confirmed HIV-1 RNA ≥ 1000 Copies/mL at or after 24 weeks) over time, in the overall study population and by selected characteristics of participants at study entry

Of the 278 participants, 139 (50%) experienced confirmed virologic failure at or after week 24 of follow-up. Figure 1 shows the cumulative proportion experiencing virologic failure by time. Most (103 of 139) of the failures occurred at week 24: the estimated proportion failing at that time was 39% (95% confidence interval [CI] 33%, 45%). The remaining 36 participants who experienced virologic failure achieved suppression below 1000 copies/mL but then experienced virologic failure after week 24. The estimated cumulative proportion failing increased with time, from 39% at week 24 to 48%, 53% and 60% at weeks 48, 72 and 96. Conversely, an estimated 40% achieved suppression below 1000 copies/mL through to 96 weeks (95% CI 33%, 49%).

Figure 1 also shows the cumulative proportion experiencing virologic failure over time by sex; age, HIV-1 RNA, CD4 count, duration of prior ART at study entry; NRTI resistance at screening; and randomized adherence support intervention. There was significant variation in time to virologic failure among the categories of each of these variables except for the randomized comparison of CPI + SOC versus SOC adherence support. Based on visual inspection of Fig. 1, categories of some variables were combined for further analysis where the distribution of time of virologic failure appeared very similar: for age, the revised categorization used was < 30 versus ≥ 30 years; for HIV-1 RNA, < 10,000 versus ≥ 10,000 copies/mL; for CD4 count, < 200 versus ≥ 200 cells/mm^3^; and for duration of ART, < 10 versus ≥ 10 years. Using these revised categories, Table 2 shows descriptive statistics for the proportion observed with virologic failure during follow-up, as well as estimated hazards ratios from univariable and multivariable proportional hazards models. In the multivariable analysis, the strongest and significant predictors of virologic failure were higher HIV-1 RNA at study entry (adjusted hazard ratio [aHR]: 2.20 for ≥ 10,000 versus < 10,000 copies/mL), lower age at study entry (aHR: 1.96 for < 30 versus ≥ 30 years), NRTI resistance at screening (aHR: 1.74 for those with versus without resistance), lower CD4 count at study entry (aHR: 1.73 for < 200 versus ≥ 200 cells/mm^3^), and shorter duration of ART (aHR 1.62 for < 10 versus ≥ 10 years). Female participants had somewhat higher risk of virologic failure than male participants (aHR: 1.30) though this was not statistically significant (p = 0.16). Although the estimated hazard ratio for CPI + SOC versus SOC was in the direction of a small benefit of CPI (aHR: 0.80), it was not statistically significant (p = 0.45). We further evaluated whether there was effect modification between each possible pair of variables included in the multivariable model and found no significant evidence (p > 0.05 for all interaction terms). We also found no significant evidence that the PI taken (LPV or ATV) was predictive when added to the model or that any of the associations were modified by which PI was taken. Similarly, with the caveat of limited power, there was no significant evidence that country was predictive of virologic failure when added to the multivariable model or that any of the associations varied among countries. Sensitivity analyses using thresholds of ≥ 50 copies/mL and ≥ 200 copies/mL instead of ≥ 1000 copies/mL to define virologic failure showed the same five variables as the strongest predictors though the hazard ratios for both HIV-1 RNA and CD4 count at study entry showed weaker associations as the threshold used decreased from 1000 to 50 copies/mL (Additional file 1: Tables S2 and S3).

**Table 2:** Virological failure (confirmed HIV-1 RNA ≥ 1000 Copies/mL at or after week 24) by selected characteristics of participants and randomized adherence support intervention

Variable	Categories	N (%)	N (%) with virologic failure	Hazard ratio (95% CI), unadjusted	Hazard ratio (95% CI) adjusted for other variables shown
Sex	Female	155	87 (56%)	1.52 (1.07, 2.15)	1.30 (0.90, 1.86)
	Male	123	52 (42%)	Reference	
Age at study entry (years)	< 30	46	35 (76%)	2.14 (1.45, 3.17)	1.96 (1.31, 2.94)
	≥ 30	232	104 (45%)	Reference	
HIV-1 RNA at study entry (copies/mL)	≥ 10,000	170	104 (61%)	2.61 (1.78, 3.84)	2.20 (1.46, 3.32)
	< 10,000	108	35 (32%)	Reference	
CD4 count at study entry (cells/mm^3^)	< 200	154	94 (61%)	2.15 (1.51, 3.08)	1.73 (1.18 2.53)
	≥ 200	124	45 (36%)	Reference	
Resistance to any NRTI at screening	Yes	104	58 (56%)	1.49 (1.06, 2.10)	1.74 (1.22, 2.48)
	No	174	81 (47%)	Reference	
Duration of ART prior to study entry (years)	< 10	210	116 (55%)	1.90 (1.21, 2.98)	1.62 (1.03, 2.56)
	≥ 10	68	23 (34%)	Reference	
Randomized Adherence Support Intervention	CPI + SOC	128	58 (45%)	0.78 (0.55, 1.11)	0.80 (0.56, 1.14)
	SOC	132	71 (54%)	Reference	
	Site didn’t participate in randomization	18	10 (56%)	0.88 (0.45, 1.72)	0.82 (0.42, 1.63)

**Fig. 2:**
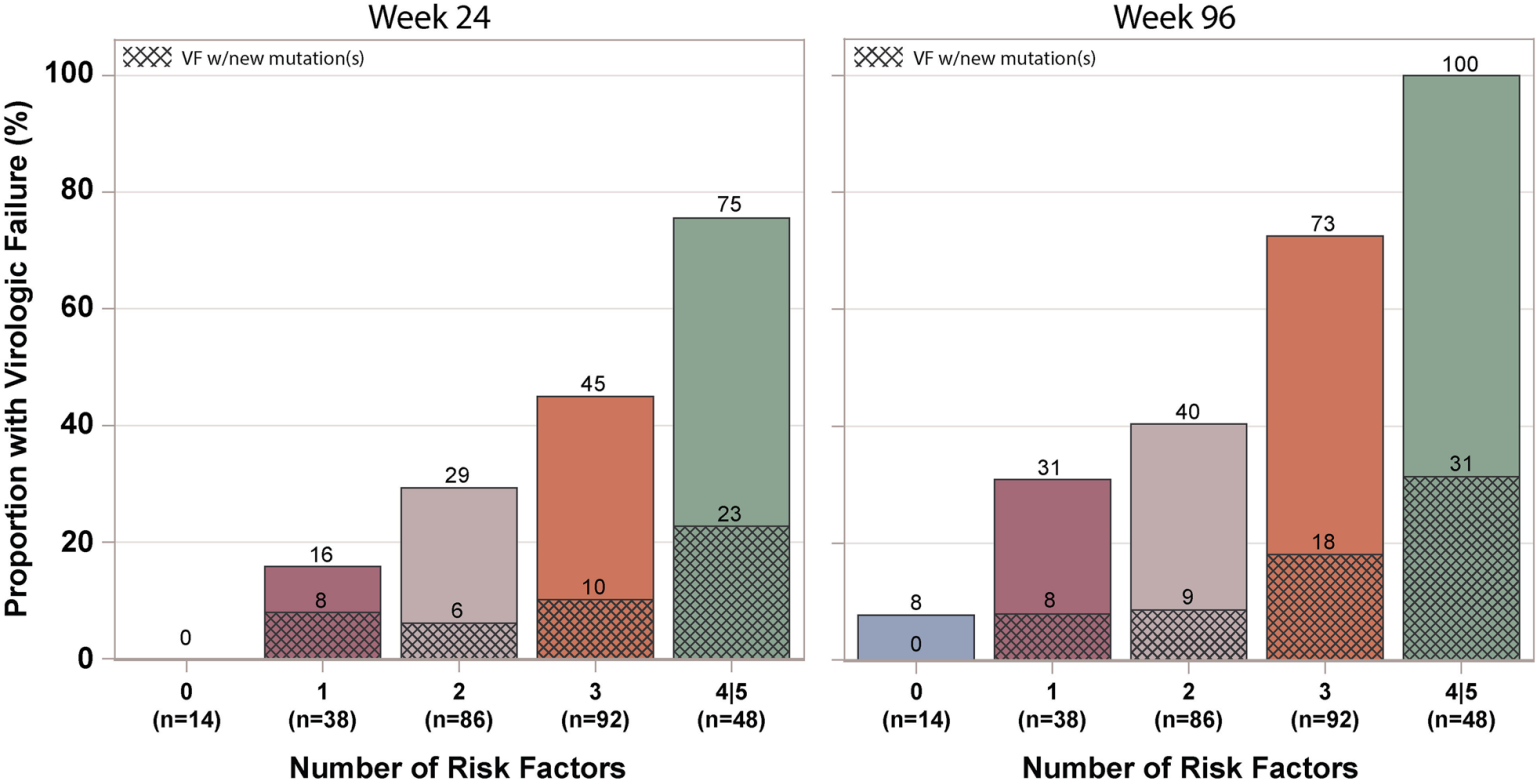
Estimated Cumulative proportion of participants with virologic failure (confirmed HIV-1 RNA ≥ 1000 Copies/mL At or after 24 weeks) at Weeks 24 and 96 by Number of Risk Factors for Virologic Failure a Participant Had**. **Bars Show Breakdown of Those With Versus Without New NRTI and/or PI Mutations. Risk Factors Were Age at Study Entry < 30 Years, HIV-1 RNA at Study Entry ≥ 10,000 Copies/mL, CD4 Count at Study Entry < 2000 Cells/mm^3^, Any NRTI Resistance Identified at Study Screening, and Duration of Prior ART at Study Entry < 10 Years

Overall, therefore, participants of age < 30 years, participants with uncontrolled HIV disease at study entry as measured by HIV-1 RNA ≥ 10,000 copies/mL or CD4 count < 200 cells/mm^3^, participants with NRTI resistance at screening, and participants on ART for < 10 years had significantly higher risk of virologic failure during follow-up. As a simple illustration of the variation in risk of virologic failure in the study population, we categorized each participant according to the number of these risk factors they had, giving a score between 0 and 5. Figure 2 shows the estimated cumulative proportion of participants experiencing virologic failure at weeks 24 and 96 by this score (as only six participants had all five risk factors and so a score of 5, participants with scores of 4 and 5 are grouped together). There was a strong trend in cumulative proportion with virologic failure at week 24 by number of risk factors varying from 0% for a score of 0 to 16%, 29%, 45% and 75% for scores of 1, 2, 3 and 4/5; and at week 96, varying from 8% for a score of 0 to 31%, 40%, 73% and 100% for scores of 1, 2, 3 and 4/5.

We also evaluated whether self-reported adherence at weeks 12 and 24 was predictive of virologic failure. Among the 180 participants reporting taking ≥ 90% of their doses in the month prior to study visits at both weeks 12 and 24, 88 (49%) experienced virologic failure. In comparison, among the 98 participants reporting taking < 90% of their doses at either or both of weeks 12 and 24, 51 (52%) experienced virologic failure. The difference was not statistically significant in univariable analysis (p = 0.15) or when added to the multivariable model (p = 0.17).

Of the 139 participants who experienced virologic failure, 134 had a resistance genotype available at virologic failure, including 36 who developed new NRTI or PI resistance mutations compared with the genotype obtained during screening for study entry (Additional file 1: Table S4). Thirty-one participants (11%) had new NRTI resistance mutations and seven (3%) had new PI resistance mutations which were not present at screening (including two who had both new NRTI and new PI resistance mutations). Twenty-six of the 36 participants developed new resistance mutations to drugs in the regimen being taken. This included high-level resistance to 3TC/FTC (17 participants), to the other NRTI being taken (two participants, one of whom also developed high-level resistance to 3TC/FTC), and to the PI being taken (one participant). Assuming that the five participants without a genotype at virologic failure had no new NRTI or PI resistance mutations, 13% of the 278 participants experienced virologic failure with new NRTI or PI resistance mutations. The same proportion of participants (13%) with versus without NRTI resistance at screening developed new resistance mutations.

Figure 2 also shows the breakdown of participants experiencing virologic failure by number of risk factors for failure between those with new NRTI or PI resistance mutations (shaded part of bar) and those without new NRTI or PI resistance mutations (unshaded part of bar). There was a significant association over time of cumulative proportion experiencing virologic failure without new mutations and risk score (p < 0.001). Despite the small number of participants experiencing virologic failure with new resistance mutations, there was also a significant association over time of cumulative proportion experiencing virologic failure with new resistance mutations and risk score (p = 0.039).

## Discussion

Increasing numbers of people living with HIV in LMICs have experienced treatment failure on NNRTI- and PI-based ART [1]. In a systematic review of second-line ART failure in sub-Saharan Africa [10], the second-line failure rate among 18,550 people was 15 per 100 person years. In reports from clinical centers where HIV-1 genotyping had been performed in these patients, wildtype genotypes were observed in 12–58% of tested individuals [11–14]. Consistent with this, in genotyping at screening for the A5288 study of people from multiple LMICs failing second-line ART and hence being considered for possible third-line ART, approximately half (278, 51%) of 545 participants had no resistance to the PI they were taking and had no or minimal resistance to NRTIs and so continued their PI-based regimen. Among these 278 participants, we found that an estimated 39% continued to experience confirmed virologic failure (HIV-1 RNA ≥ 1000 copies/mL) at 24 weeks after study entry, increasing to 60% at 96 weeks, though conversely an estimated 61% achieved suppression at 24 weeks and 40% continued to be suppressed at 96 weeks.

Given the importance of maintaining virologic suppression for reducing risk of HIV disease progression and transmission, we undertook an exploratory analysis to evaluate whether there might be factors predictive of virologic failure among people failing second-line ART with limited NRTI resistance who continued on their PI-based regimen. We identified five factors that were strong and statistically significant predictors of increased risk in both univariable and multivariable modeling: HIV-1 RNA ≥ 10,000 copies/mL, age < 30 years, NRTI resistance, CD4 count < 200 cells/mm^3^, and duration of ART < 10 years. A simple scoring system which counted how many of these five factors were present in each individual was highly predictive, with an estimated risk of virologic failure by 96 weeks ranging from 8% for a score of 0 to 31%, 40%, 73% and 100% for scores of 1, 2, 3 and 4/5. Although the performance of such a scoring system needs validating in independent datasets, if confirmed it would provide a simple mechanism for identifying individuals (e.g. with a score of 0 or 1) who might be able to successfully achieve and maintain re-suppression on their PI-based ART.

Among the five factors identified, several might reflect issues with adherence to ART. Both HIV-1 RNA ≥ 10,000 copies/mL and CD4 count < 200 cells/mm^3^ might reflect uncontrolled HIV disease due to long-term incomplete adherence, particularly in environments in which HIV-1 RNA monitoring may not have been present or may have been limited. Treatment guidelines recognize adherence challenges in young adults [15]. Experiencing virologic failure on second-line ART with shorter duration of ART (< 10 years) versus longer may reflect increased challenges with adherence. In contrast, the presence of NRTI resistance, albeit limited as individuals were only allowed to continue their PI-based ART in the A5288 study with resistance to one NRTI in their regimen, may reflect decreased potency of the regimen being continued.

In a systematic review, the value of self-reported adherence for predicting virologic outcome was mixed across studies [16]. In our study, participant self-report of missed doses at weeks 12 and 24 did not predict subsequent virologic failure in either univariable or multivariable analysis, despite the fact that retention in follow-up among participants in our study was extremely high. Improving adherence is challenging as illustrated by the limited effect in the A5288 study of adding a two-way cellphone adherence support intervention to sites’ standard of care adherence support [8]: the observed proportion experiencing virologic failure was reduced from 54% for SOC to 45% for CPI + SOC. An interplay of psycho-social, cultural, personal and public health infrastructure can impact treatment adherence [8]. Further research is needed to develop and evaluate other approaches for improving adherence in this population. Simpler antiretroviral regimens including long-acting options might also be needed to improve outcomes.

Compared with men, women in our study also had a significantly increased risk of virologic failure when continuing their second-line regimen in univariable analysis but the association was reduced and not statistically significant in multivariable analysis. This is consistent with our previous findings in the broader A5288 study population, which may have been associated with increased issues with tolerability to PI-based regimens in women than men, though there may be other factors such as reduced drug availability in women than men [17].

Continuation of second-line ART following virologic failure raises the possibility of development of new resistance mutations. During a median 72 weeks of follow-up, 13% of the study population developed new resistance mutations. We found that the risk of virologic failure with new resistance mutations was also associated with our simple scoring based on the five risk factors identified. Development of new NRTI resistance mutations was more common than new PI resistance mutations, and the latter were generally observed among those with pre-existing NRTI resistance at screening for entry into the study. Not all new resistance mutations observed at virologic failure were mutations indicative of resistance to the drugs being taken, suggesting naturally occurring polymorphisms or re-emergence of mutations conferring resistance to drugs a participant had previously taken (or transmitted resistant virus).

A strength of this study was large number of enrolled participants from diverse urban sites in 10 LMICs. On the other hand, our findings come from a clinical trial and so may not generalize to general clinical practice or to rural populations. In particular, retention in care was very high in our study and potentially higher than might be achieved in practice. The clinical trial included enrollment between 2013 and 2015 and so it is possible that predictors of outcome have changed with time. Participants in our study remained on LPV/r- or ATV/r-based regimens which, though still widely used, may not be as well tolerated as newer generation NNRTIs, PIs (including ritonavir-boosted darunavir) and integrase inhibitors [18] as indicated by the high occurrence of adverse events noted in these participants [7]. However, in the clinical trial, participants in Cohort A were not randomized to alternative antiretroviral regimens and so we cannot assess whether participants would have had better outcomes if they had been started on novel antiretrovirals with better tolerance, more convenient once daily formulations and greater potency. Whether our simple score might be used to predict outcomes when continuing such novel regimens following virologic failure without substantial resistance would need to be evaluated in further studies. Lastly, results from our study are only applicable to settings in which resources are available for genotypic resistance testing to identify participants without PI resistance and with limited NRTI resistance, for whom staying on PI-based therapy might be a reasonable treatment option.

## Conclusion

In conclusion, in this analysis, we identified factors that predict whether further virologic failure is likely or whether virologic re-suppression may be achieved and maintained when continuing PI-based ART among participants experiencing failure on that regimen with no PI resistance and no or limited NRTI resistance. The simple scoring system based on these factors merits further evaluation to assess whether its use coupled with novel adherence support strategies and newer antiretroviral regimens might enhance outcomes in this population.

## Supplementary Information

**Additional file 1: Fig. S1.** Flow Chart Describing the Construction of the Study Population for this Report. **Table S1.** Resistance Profiles of Participants in the Study Population at Screening to Study Entry. **Table S2.** Results from Proportional Hazards Model When Virological Failure is Defined as Having Two Successive HIV-1 RNA Measurements ≥50 Copies/mL At or After Week 24 (Instead of Two Successive HIV-1 RNA Measurements ≥1000 Copies/mL As in Table 2 of the Manuscript). **Table S3.** Results from Proportional Hazards Model When Virological Failure is Defined as Having Two Successive HIV-1 RNA Measurements ≥200 Copies/mL At or After Week 24 (Instead of Two Successive HIV-1 RNA Measurements ≥1000 Copies/mL As in Table 2 of the Manuscript). **Table S4.** Changes in Nucleoside Reverse Transcriptase Inhibitor (NRTI) and Protease Inhibitor (PI) Resistance Profiles Between Study Screening and Virologic Failure for the 36 Participants with New NRTI- and/or PI-Associated Resistance Mutations at Virologic Failure.

## Data Availability

All of the data generated in the study are presented in the tables and figures in the manuscript and supplementary materials.

## References

[1] Gupta A, Junerja S, Vitoria M (2016). Projected uptake of new antiretroviral (ARV) medicines in adults in low- and middle-income countries; a forecast analysis 2015–2025. PLoS ONE.

[2] World Health Organization. Update of recommendations on first-and second-line antiretroviral regimens. Geneva, Switzerland: World Health Organization; 2019 (WHO/CDS/HIV/19.15). Licence: CC BY-NC-SA 3.0 IGO.https://apps.who.int/iris/bitstream/handle/10665/325892/WHO-CDS-HIV-19.15-eng.pdf. Accessed 11 Aug 2020.

[3] Cambiano V, Bertagnolio S, Jordan MR (2014). Predicted levels of HIV drug resistance: potential impact of expanding diagnosis, retention, and eligibility criteria for antiretroviral therapy initiation. AIDS.

[4] Wallis CL, Hughes MD, Ritz J (2019). Diverse HIV-1 drug resistance profiles at screening for ACTG A5288: a study of people experiencing virologic failure on second-line ART in resource limited settings. Clin Infect Dis.

[5] Khan S, Das M, Andries A (2014). Second-line failure and first experience with third-line antiretroviral therapy in Mumbai, India. Global Health Action.

[6] Moorhouse M, Maartens G, Venter WD (2019). Third-line antiretroviral therapy program in the south african public sector: cohort description and virological outcomes. J Acquir Immune Defic Syndr.

[7] Grinsztejn B, Hughes MD, Ritz J (2019). Third-line antiretroviral therapy in low-income and middle-income countries (ACTG A5288): a prospective strategy study. Lancet HIV.

[8] Gross R, Ritz J, Hughes MD (2019). Two-way mobile phone intervention compared to standard-of-care adherence support after second-line antiretroviral failure: a multinational, randomized controlled trial. Lancet Digit Health.

[9] Liu TF, Shafer RW (2006). Web resources for HIV type 1 genotypic-resistance test interpretation. Clin Infect Dis.

[10] Edessa D, Sisay M, Asefa F (2019). Second-line HIV treatment failure in sub-saharan Africa: a systematic review and meta-analysis. Plus One.

[11] Ndahimana JD, Riedel DJ, Mahayimpundu R (2016). HIV drug resistance mutations among patients failing second-line antitetroviral therapy in Rwanda. Antivir Ther.

[12] Maiga AI, Fofana DB, Cisse M (2012). Characterization of HIV-1 antiretroviral drug resistance after second-line treatment failure in Mali, a limited-resources setting. J Antimicrob Chemother.

[13] Fily F, Ayikobua E, Ssemwanga D (2018). HIV-1 drug resistance testing at second-line regimen failure in Arua, Uganda: avoiding unnecessary switch to an empiric third-line. Trop Med Int Health.

[14] Sawadogo S, ShiningavamweA Roscoe C (2018). Human immunodeficiency virus-1 drug resistance patternsamong adult patients failing second-line protease inhibitor-containing regimensin Namibia, 2010–2015. Open Forum Infect Dis.

[15] World Health Organization (2013). HIV and adolescents: guidance for HIV testing and counselling and care for adolescents living with HIV: recommendations for a public health approach and considerations for policy-makers and managers.

[16] Almeida-Brasil CC, Moodie EE, Cardoso TS, Nascimento ED, Ceccato MDGB (2019). Comparison of the predictive performance of adherence measures for virologic failure detection in people living with HIV: a systematic review and pairwise meta-analysis. AIDS Care.

[17] Godfrey C, Hughes MD, Ritz J (2020). Sex differences in outcomes for individuals presenting for third-line antiretroviral therapy. J Acquir Immune Defic Syndr.

[18] Domingo P, Mateo MG, Guiterrez MDM (2018). Tolerability of current antiretroviral single-tablet regimens. AIDS Rev.

