# Supplementary material for: Predictors of virologic outcome among people living with HIV who continue a protease inhibitor-based antiretroviral regimen following virologic failure with no or limited resistance
Source: AIDS Res Ther. 2023 Jan 5;20:3. doi: 10.1186/s12981-022-00494-9 (PMC9814171; doi:10.1186/s12981-022-00494-9)
Supplement: Supplementary file 1 — Additional file 1: Fig. S1. Flow Chart Describing the Construction of the Study Population for this Report. Table S1. Resistance Profiles of Participants in the Study Population at Screening to Study Entry. Table S2. Results from Proportional Hazards Model When Virological Failure is Defined as Having Two Successive HIV-1 RNA Measurements ≥50 Copies/mL At or After Week 24 (Instead of Two Successive HIV-1 RNA Measurements ≥1000 Copies/mL As in Table 2 of the Manuscript). Table S3. Results from Proportional Hazards Model When Virological Failure is Defined as Having Two Successive HIV-1 RNA Measurements ≥200 Copies/mL At or After Week 24 (Instead of Two Successive HIV-1 RNA Measurements ≥1000 Copies/mL As in Table 2 of the Manuscript). Table S4. Changes in Nucleoside Reverse Transcriptase Inhibitor (NRTI) and Protease Inhibitor (PI) Resistance Profiles Between Study Screening and Virologic Failure for the 36 Participants with New NRTI- and/or PI-Associated Resistance Mutations at Virologic Failure. [file 12981_2022_494_MOESM1_ESM.docx]

**Predictors of Virologic Outcome Among People Living with HIV Who Continue A Protease Inhibitor-based Antiretroviral Regimen Following Virologic Failure With No or Limited Resistance**

Robert A. Salata, Beatriz Grinsztejn, Justin Ritz, Ann C. Collier, Evelyn Hogg, Robert Gross, Catherine Godfrey, Nagalingeswaran Kumarasamy, Cecilia Kanyama, John W. Mellors, Carole L. Wallis, Michael D. Hughes for the ACTG A5288 Study Team

**Additional file**

**Figure S1. Flow Chart Describing the Construction of the Study Population for this Report**

**
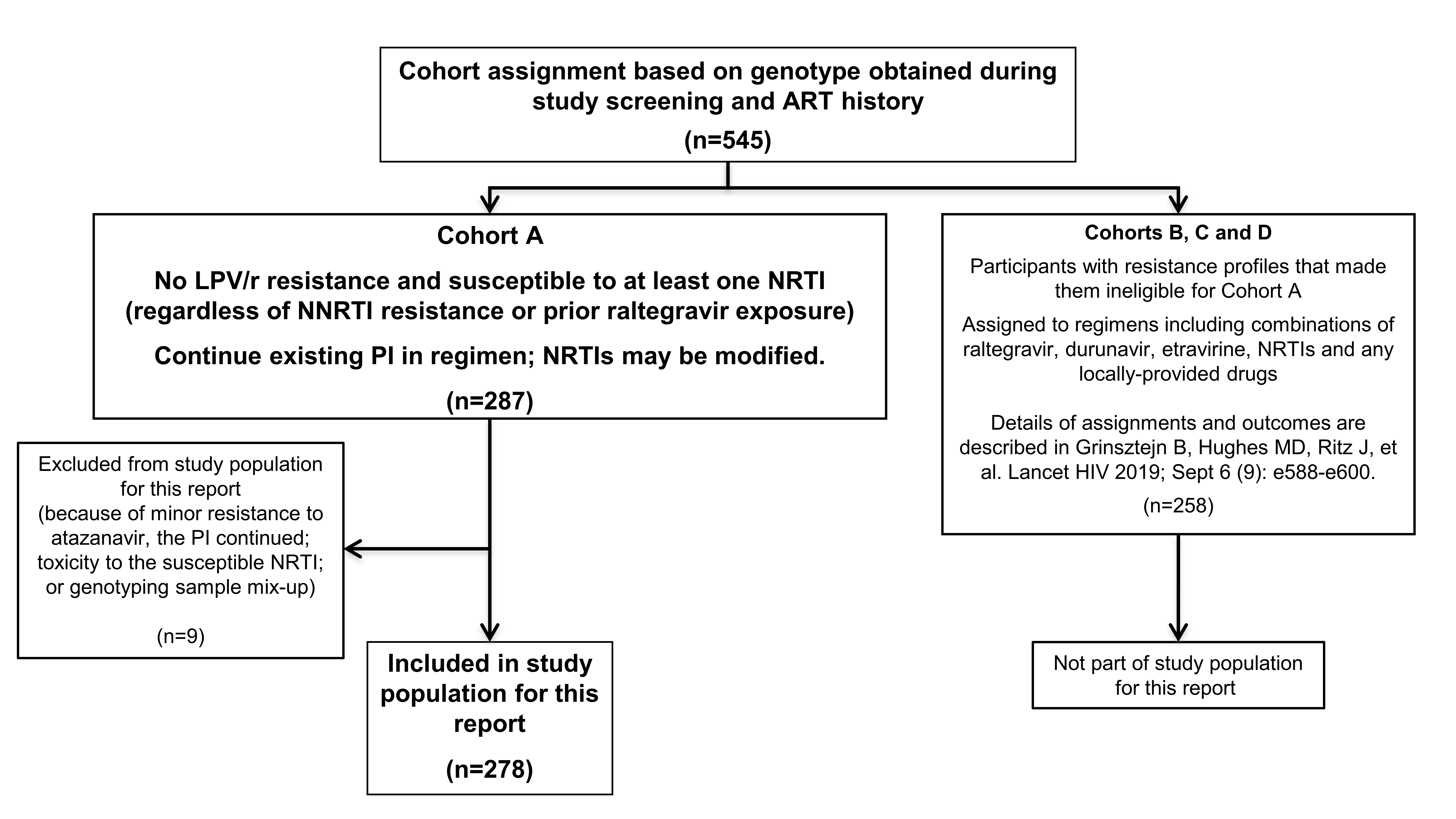
**

**Table S1. Resistance Profiles of Participants in the Study Population at Screening to Study Entry**

**Table S2. Results from Proportional Hazards Model When Virological Failure is Defined as Having Two Successive HIV-1 RNA Measurements ≥50 Copies/mL At or After Week 24 (Instead of Two Successive HIV-1 RNA Measurements ≥1000 Copies/mL As in Table 2 of the Manuscript)**

| **Variable** | **Categories** | **N (%)** | **N (%) with virologic failure** | **Hazard Ratio  (95% CI), unadjusted** | **Hazard Ratio  (95% CI) adjusted for other variables shown** |
| --- | --- | --- | --- | --- | --- |
| **Sex** | **Female** | 155 | 111 (72%) | 1.30 (0.97, 1.74) | 1.24 (0.91, 1.70) |
|  | **Male** | 123 | 79 (64%) | Reference | |
| **Age at study entry (years)** | **<30** | 46 | 43 (93%) | 2.12 (1.47, 3.05) | 2.09 (1.41, 3.08) |
|  | **≥30** | 232 | 147 (63%) | Reference | |
| **HIV-1 RNA at study entry (copies/mL)** | **≥10,000** | 170 | 126 (74%) | 1.71 (1.26, 2.33) | 1.63 (1.16, 2.29) |
|  | **<10,000** | 108 | 64 (59%) | Reference | |
| **CD4 count at study entry (cells/mm^3^)** | **<200** | 154 | 115 (75%) | 1.63 (1.21, 2.20) | 1.38 (0.99, 1.91) |
|  | **≥200** | 124 | 75 (60%) | Reference | |
| **Resistance to any NRTI at screening** | **Yes** | 104 | 77 (74%) | 1.67 (1.23, 2.27) | 1.88 (1.36, 2.60) |
|  | **No** | 174 | 113 (65%) | Reference | |
| **Duration of ART prior to study entry (years)** | **<10** | 210 | 155 (74%) | 1.88 (1.30, 2.74) | 1.70 (1.17, 2.49) |
|  | **≥10** | 68 | 35 (51%) | Reference | |
| **Randomized Adherence Support Intervention** | **CPI+SOC** | 128 | 88 (69%) | 1.04 (0.77, 1.42) | 1.12 (0.81, 1.54) |
|  | **SOC** | 132 | 87 (66%) | Reference | |
|  | **Site didn’t participate in randomization** | 18 | 15 (83%) | 1.21 (0.69, 2.13) | 1.23 (0.68, 2.24) |

**Table S3. Results from Proportional Hazards Model When Virological Failure is Defined as Having Two Successive HIV-1 RNA Measurements ≥200 Copies/mL At or After Week 24 (Instead of Two Successive HIV-1 RNA Measurements ≥1000 Copies/mL As in Table 2 of the Manuscript)**

| **Variable** | **Categories** | **N (%)** | **N (%) with virologic failure** | **Hazard Ratio  (95% CI), unadjusted** | **Hazard Ratio  (95% CI) adjusted for other variables shown** |
| --- | --- | --- | --- | --- | --- |
| **Sex** | **Female** | 155 | 99 (64%) | 1.25 (0.91, 1.70) | 1.13 (0.82, 1.57) |
|  | **Male** | 123 | 70 (57%) | Reference | |
| **Age at study entry (years)** | **<30** | 46 | 40 (87%) | 2.06 (1.42, 2.97) | 1.94 (1.32, 2.85) |
|  | **≥30** | 232 | 129 (56%) | Reference | |
| **HIV-1 RNA at study entry (copies/mL)** | **≥10,000** | 170 | 119 (70%) | 2.08 (1.49, 2.90) | 1.86 (1.29, 2.67) |
|  | **<10,000** | 108 | 50 (46%) | Reference | |
| **CD4 count at study entry (cells/mm^3^)** | **<200** | 154 | 106 (69%) | 1.85 (1.35, 2.54) | 1.53 (1.09, 2.16) |
|  | **≥200** | 124 | 63 (51%) | Reference | |
| **Resistance to any NRTI at screening** | **Yes** | 104 | 69 (66%) | 1.53 (1.11, 2.10) | 1.78 (1.28, 2.49) |
|  | **No** | 174 | 100 (57%) | Reference | |
| **Duration of ART prior to study entry (years)** | **<10** | 210 | 137 (65%) | 1.69 (1.15, 2.49) | 1.46 (0.98, 2.17) |
|  | **≥10** | 68 | 32 (47%) | Reference | |
| **Randomized Adherence Support Intervention** | **CPI+SOC** | 128 | 74 (58%) | 0.91 (0.66, 1.25) | 0.98 (0.70, 1.36) |
|  | **SOC** | 132 | 81 (61%) | Reference | |
|  | **Site didn’t participate in randomization** | 18 | 14 (78%) | 1.14 (0.64, 2.03) | 1.14 (0.63, 2.07) |

**Table S4. Changes in Nucleoside Reverse Transcriptase Inhibitor (NRTI) and Protease Inhibitor (PI) Resistance Profiles Between Study Screening and Virologic Failure for the 36 Participants with New NRTI- and/or PI-Associated Resistance Mutations at Virologic Failure**

|  | **Change in Resistance Profile to Regimen Received** | | |  | **Resistance Interpretation for NRTIs in Regimen** | | | | | **Resistance Interpretation for PI in Regimen** | | |
| --- | --- | --- | --- | --- | --- | --- | --- | --- | --- | --- | --- | --- |
| **ID** | **3TC/FTC** | **Other NRTI** | **PI** | **Evaluation Time** | **NRTI Mutations** | **3TC/FTC** | **TDF** | **ZDV** | **ABC** | **PI Mutations** | **ATV/r** | **LPV/r** |
| 1 | New High-level | New High-level |  | Screening | - | Susceptible | Susceptible |  |  | - |  | Susceptible |
|  | Resistance | Resistance |  | Failure | M41LM,E44DE,D67DN,L74LV,V118IV,M184V,L210LW,T215SY,N348IN | High-level | High-level |  |  | - |  | Susceptible |
| 2 | New High-level | New Low-level |  | Screening | - | Susceptible |  |  | Susceptible | - | Susceptible |  |
|  | Resistance | Resistance |  | Failure | M184V | High-level |  |  | Low-level | - | Susceptible |  |
| 3 | New High-level | New Low-level |  | Screening | - | Susceptible | Susceptible |  |  | - |  | Susceptible |
|  | Resistance | Resistance |  | Failure | M41L,M184V,T215Y | High-level | Low-level |  |  | - |  | Susceptible |
| 4 | New High-level  Resistance |  | New Potential Low-level | Screening | T69NT | Susceptible | Susceptible |  |  | L10V,A71T | Susceptible |  |
|  |  |  | Resistance | Failure | D67N,M184V | High-level | Susceptible |  |  | L10V,L23I,F53IL,  A71IT | Potential low-level |  |
| 5 | New High-level |  |  | Screening | D67N,T69DN,K70R | Susceptible | Low-level |  |  | - | Susceptible |  |
|  | Resistance |  |  | Failure | D67N,T69DN,K70R,M184V,K219KQ | High-level | Potential low-level |  |  | - | Susceptible |  |
| 6 | New High-level |  |  | Screening | - | Susceptible | Susceptible |  |  | - | Susceptible |  |
|  | Resistance |  |  | Failure | M184V | High-level | Susceptible |  |  | - | Susceptible |  |
| 7 | New High-level |  |  | Screening | - | Susceptible | Susceptible |  |  | - | Susceptible |  |
|  | Resistance |  |  | Failure | M184V | High-level | Susceptible |  |  | - | Susceptible |  |
| 8 | New High-level |  |  | Screening | - | Susceptible | Susceptible |  |  | L10V,T74S |  | Susceptible |
|  | Resistance |  |  | Failure | M184V | High-level | Susceptible |  |  | L10V,T74S |  | Susceptible |
| 9 | New High-level |  |  | Screening | - | Susceptible | Susceptible |  |  | - |  | Susceptible |
|  | Resistance |  |  | Failure | M184MV | High-level | Susceptible |  |  | - |  | Susceptible |
| 10 | New High-level |  |  | Screening | - | Susceptible | Susceptible |  |  | - |  | Susceptible |
|  | Resistance |  |  | Failure | M184V | High-level | Susceptible |  |  | - |  | Susceptible |
| 11 | New High-level |  |  | Screening | E44D | Susceptible | Susceptible |  |  | - |  | Susceptible |
|  | Resistance |  |  | Failure | M184V | High-level | Susceptible |  |  | - |  | Susceptible |
| 12 | New High-level |  |  | Screening | - | Susceptible | Susceptible |  |  | - | Susceptible |  |
|  | Resistance |  |  | Failure | M184V | High-level | Susceptible |  |  | - | Susceptible |  |
| 13 | New High-level |  |  | Screening | - | Susceptible |  | Susceptible |  | - | Susceptible |  |
|  | Resistance |  |  | Failure | M184V | High-level |  | Susceptible |  | - | Susceptible |  |
| 14 | New High-level |  |  | Screening | - | Susceptible | Susceptible |  |  | - | Susceptible |  |
|  | Resistance |  |  | Failure | M184MV | High-level | Susceptible |  |  | - | Susceptible |  |
| 15 | New High-level |  |  | Screening | - | Susceptible | Susceptible |  |  | - | Susceptible |  |
|  | Resistance |  |  | Failure | M184V | High-level | Susceptible |  |  | - | Susceptible |  |
| 16 | New High-level |  |  | Screening | - | Susceptible | Susceptible |  |  | L10IV |  | Susceptible |
|  | Resistance |  |  | Failure | M184V | High-level | Susceptible |  |  | L10IV |  | Susceptible |
| 17 | New High-level |  |  | Screening | T69D | Susceptible | Susceptible |  |  | K20I |  | Susceptible |
|  | Resistance |  |  | Failure | T69D,M184MV | High-level | Susceptible |  |  | K20I |  | Susceptible |
| 18 |  | New High-level |  | Screening | - | Susceptible |  | Susceptible |  | - | Susceptible |  |
|  |  | Resistance |  | Failure | D67DN,T215NSTY | Susceptible |  | High-level |  | - | Susceptible |  |
| 19 |  | New Intermediate- |  | Screening | M184V | High-level | Susceptible |  |  | - |  | Susceptible |
|  |  | Level Resistance |  | Failure | K65R,M184V | High-level | Intermediate |  |  | - |  | Susceptible |
| 20 |  | New Low-level |  | Screening | M184MV | High-level | Susceptible |  |  | - | Susceptible |  |
|  |  | Resistance |  | Failure | K70EK,M184V | High-level | Low-level |  |  | - | Susceptible |  |
| 21 |  | New Low-level |  | Screening | M184V | High-level | Susceptible |  |  | - |  | Susceptible |
|  |  | Resistance |  | Failure | M41LM,M184V,T215F,N348I | High-level | Low-level |  |  | - |  | Susceptible |
| 22 | New Potential Low-level | New Potential Low-level |  | Screening | - | Susceptible | Susceptible |  |  | - | Susceptible |  |
|  | Resistance | Resistance |  | Failure | K70T | Potential low-level | Potential low-level |  |  | - | Susceptible |  |
| 23 |  |  | New High-level | Screening | M184V | High-level | Susceptible |  |  | L10V | Susceptible |  |
|  |  |  | Resistance | Failure | M184V | High-level | Susceptible |  |  | L10V,I50L,A71V,  G73GS,V82A | High-level |  |
| 24 |  |  | New Intermediate- | Screening | K65R,V118I,M184V | High-level |  | Susceptible |  | L10IL |  | Susceptible |
|  |  |  | Level Resistance | Failure | K65R,V118I,M184V | High-level |  | Susceptible |  | L10I,I54IV,V82A |  | Intermediate |
| 25 |  |  | New Low-level | Screening | M184V | High-level | Susceptible |  |  | - |  | Susceptible |
|  |  |  | Resistance | Failure | M184V | High-level | Susceptible |  |  | V82AV |  | Low-level |
| 26 |  |  | New Low-level | Screening | M184V,T215Y | High-level | Susceptible |  |  | - |  | Susceptible |
|  |  |  | Resistance | Failure | M184V,T215Y,N348I | High-level | Susceptible |  |  | V82A |  | Low-level |
| 27 | New NRTI and/or PI mutations identified,  but not to drugs in regimen received | | | Screening | - | Susceptible | Susceptible |  |  | - |  | Susceptible |
|  |  |  |  | Failure | L74LV | Susceptible | Susceptible |  |  | - |  | Susceptible |
| 28 | New NRTI and/or PI mutations identified,  but not to drugs in regimen received | | | Screening | - | Susceptible | Susceptible |  |  | - |  | Susceptible |
|  |  |  |  | Failure | N348IN | Susceptible | Susceptible |  |  | - |  | Susceptible |
| 29 | New NRTI and/or PI mutations identified,  but not to drugs in regimen received | | | Screening | - | Susceptible | Susceptible |  |  | - | Susceptible |  |
|  |  |  |  | Failure | T69N | Susceptible | Susceptible |  |  | - | Susceptible |  |
| 30 | New NRTI and/or PI mutations identified,  but not to drugs in regimen received | | | Screening | - | Susceptible |  | Susceptible |  | - |  | Susceptible |
|  |  |  |  | Failure | T69N | Susceptible |  | Susceptible |  | - |  | Susceptible |
| 31 | New NRTI and/or PI mutations identified,  but not to drugs in regimen received | | | Screening | - | Susceptible | Susceptible |  |  | - |  | Susceptible |
|  |  |  |  | Failure | T69N | Susceptible | Susceptible |  |  | - |  | Susceptible |
| 32 | New NRTI and/or PI mutations identified,  but not to drugs in regimen received | | | Screening | V118IV | Susceptible | Susceptible |  |  | L10I | Susceptible |  |
|  |  |  |  | Failure | V118IV | Susceptible | Susceptible |  |  | L10I,A71AT | Susceptible |  |
| 33 | New NRTI and/or PI mutations identified,  but not to drugs in regimen received | | | Screening | - | Susceptible | Susceptible |  |  | A71T | Susceptible |  |
|  |  |  |  | Failure | - | Susceptible | Susceptible |  |  | L10V,A71T | Susceptible |  |
| 34 | New NRTI and/or PI mutations identified,  but not to drugs in regimen received | | | Screening | M184V | High-level | Susceptible |  |  | - |  | Susceptible |
|  |  |  |  | Failure | K70KQR,M184V | High-level | Susceptible |  |  | - |  | Susceptible |
| 35 | New NRTI and/or PI mutations identified,  but not to drugs in regimen received | | | Screening | M184V,T215FV | High-level | Susceptible |  |  | L10V | Susceptible |  |
|  |  |  |  | Failure | M184V,T215AITV | High-level | Susceptible |  |  | L10V | Susceptible |  |
| 36 | New NRTI and/or PI mutations identified,  but not to drugs in regimen received | | | Screening | M184V | High-level | Susceptible |  |  | L10V |  | Susceptible |
|  |  |  |  | Failure | M41L,M184V | High-level | Susceptible |  |  | L10V |  | Susceptible |

*The identification of new resistance-associated mutations was defined as the development of a mutation, not present at screening, identified and scored by the Stanford HIV database version 6.2 algorithm (https://hivdb.stanford.edu/)*
